# Quantitative Comparison of Knowledge-Based and Manual Intensity Modulated Radiation Therapy Planning for Nasopharyngeal Carcinoma

**DOI:** 10.3389/fonc.2020.551763

**Published:** 2021-01-07

**Authors:** Jiang Hu, Boji Liu, Weihao Xie, Jinhan Zhu, Xiaoli Yu, Huikuan Gu, Mingli Wang, Yixuan Wang, ZhenYu Qi

**Affiliations:** ^1^ Department of Radiation Oncology, Sun Yat-sen University Cancer Center, State Key Laboratory of Oncology in South China, Collaborative Innovation Center for Cancer Medicine, Guangdong Key Laboratory of Nasopharyngeal Carcinoma Diagnosis and Therapy, Guangzhou, China; ^2^ Sun Yat-sen Memory Hospital, Guangzhou, China

**Keywords:** knowledge-based planning, intensity modulated radiation therapy, nasopharyngeal carcinoma, plan quality, dose volume histogram prediction model

## Abstract

**Background and purpose:**

To validate the feasibility and efficiency of a fully automatic knowledge-based planning (KBP) method for nasopharyngeal carcinoma (NPC) cases, with special attention to the possible way that the success rate of auto-planning can be improved.

**Methods and materials:**

A knowledge-based dose volume histogram (DVH) prediction model was developed based on 99 formerly treated NPC patients, by means of which the optimization objectives and the corresponding priorities for intensity modulation radiation therapy (IMRT) planning were automatically generated for each head and neck organ at risk (OAR). The automatic KBP method was thus evaluated in 17 new NPC cases with comparison to manual plans (MP) and expert plans (EXP) in terms of target dose coverage, conformity index (CI), homogeneity index (HI), and normal tissue protection. To quantify the plan quality, a metric was applied for plan evaluation. The variation in the plan quality and time consumption among planners was also investigated.

**Results:**

With comparable target dose distributions, the KBP method achieved a significant dose reduction in critical organs such as the optic chiasm (p<0.001), optic nerve (p=0.021), and temporal lobe (p<0.001), but failed to spare the spinal cord (p<0.001) compared with MPs and EXPs. The overall plan quality evaluation gave mean scores of 144.59±11.48, 142.71±15.18, and 144.82±15.17, respectively, for KBPs, MPs, and EXPs (p=0.259). A total of 15 out of 17 KBPs (i.e., 88.24%) were approved by our physician as clinically acceptable.

**Conclusion:**

The automatic KBP method using the DVH prediction model provided a possible way to generate clinically acceptable plans in a short time for NPC patients.

## Introduction

Intensity modulation radiation therapy (IMRT) has become a major treatment modality for nasopharyngeal carcinoma (NPC). Compared with traditional two-dimensional radiotherapy and three-dimensional conformal radiotherapy, IMRT uses inverse planning algorithms to generate fields of varied beam intensity which allows a higher radiation dose to be delivered to the tumor while minimizing exposure to the surrounding healthy organs (1, 2). Recent reports have proven a better 5-year overall survival, tumor local control, and fewer late toxicities for NPC patients treated with IMRT (3, 4).

Although the clinical benefits of IMRT for NPC treatment have been confirmed, a renewed concern has recently arisen on the quality of IMRT planning. Currently, IMRT planning is still a trial-and-error procedure, in which dosimetrists are required to predetermine all the starting optimization objectives for tumor targets and organs at risk (OARs), and manually adjust them during the optimization process until the desired dose distribution is achieved. This is a challenging process because the optimization objectives are usually unknown before planning and geometrical anatomy-based features vary among patients. It has already been demonstrated that the plan quality relies heavily on the experience of a dosimetrist and the time spent on a given plan (5). What is worse, the recommended IMRT quality assurance protocols can only check whether the planning parameters are correct or not, they can not verify whether the plan has an optimal dose distribution. Therefore, it is essential to explore new methods to guide planners of varied skill levels to generate high quality plans in a more efficient way.

Many efforts have been made to offer a clearer directionality during IMRT planning by utilizing both patient anatomical information and past planning experience. Early exploration was conducted by Wu et al. (6, 7) who proposed an information retrieval method which utilized an overlap volume histogram to find similar plans of previous patients in a database as initial planning goals to guide the new planning procedure. Moore et al. (8) formulized the correlation between the principle OAR mean dose and the percentage of that OAR overlapping the planning target volume (PTV) to yield a simple dose prediction model, striving to provide a quality control tool for clinical IMRT planning. Recently, more sophisticated frameworks like machine learning were introduced to create refined dose volume histogram (DVH) estimation algorithms (9, 10) and preliminary results demonstrated that such knowledge-based planning (KBP) methods helped improve plan quality and planning efficiency by integrating the prior information into the planning process (11, 12).

While the KBP method has been found to be useful in many treatment sites (12–14), a newly published work revealed that less than half of fully automatic KBP plans for NPC cases can satisfy the clinical acceptance criteria (15). This is mainly due to the proximity of neighboring critical structures to the tumor target so that any slight improvement in target dose coverage may also result in those structures exceeding the primary objective dose constraints. Thus the purpose of this study is to validate the suitability and efficiency of the fully automatic KBP for NPC cases, with special attention to the possible ways that the success rate of auto-planning can be improved. To quantitatively evaluate plan quality, a quality assessing tool with built-in scoring criteria was introduced. The potential benefits of combining this quality metric with estimated DVHs for quick plan quality check were discussed.

## Methods and Materials

### Prior Plan Selection

To generate the DVH prediction model, 99 prior IMRT plans for NPC patients were retrospectively selected from our institutional database. The TNM staging information is shown in **Table 1**. All patients were immobilized in the supine position with head-neck-shoulder thermoplastic masks. A 9 co-planar beam IMRT plan with a collimator angle fixed at 0° was designed for each case by a senior physicist using the Eclipse treatment planning system (version 11.0, Varian Medical Systems, Palo Alto, CA). The dose prescription was set to 70 Gy in 30 fractions to the planning gross target volume (PGTV), 60 Gy in 30 fractions to the planning target volume (PTV1), and 54 Gy in 30 fractions to the planning target volume (PTV2). For NPC, the planning target volumes (PTV1 and PTV2) were constructed automatically by expanding the corresponding clinical target volumes (CTV1 and CTV2) in three dimensions by 3 mm, allowing for setup uncertainties. Specifically, CTV1 includes the high-risk regions of microscopic infiltration surrounding the primary gross target volume (GTV), which is defined as GTV plus a 5-10 mm margin, including the entire nasopharyngeal mucosa. CTV2 is defined as CTV1 plus a 5-10 mm margin to encompass the low-risk anatomic sites of microscopic extension. Besides, the located neck levels of the lymph nodes, and the elective neck irradiation levels are also defined as CTV2. The planning goals for tumor targets and dose constraints for the OARs were chosen according to our department protocols and national and international recommendations (16, 17). Recent follow-ups indicated that all patients were proven to have favorable prognoses with neither severe late toxicity nor treatment failure (local recurrence/distant metastasis).

**Table 1:** The 7th UICC/AJCC clinical stage information of 99 nasopharyngeal carcinoma patients.

T stage		N stage		Overall stage	
T1	5	N0	7	Stage I	2
T2	35	N1	37	Stage II	14
T3	35	N2	32	Stage III	43
T4	24	N3	23	Stage IV	40
Total	99		99		99

### Generating a KBP Plan

In this study, a mathematical framework was performed to derive DVH estimation models for head and neck OARs from high quality prior plans, similar to Zhu et al. (9). The model incorporated two major groups of anatomical features including volumetric information and spatial information, which were characterized by the minimum distance from a voxel to the PTV surface (distance-to-target histogram, DTH). The DTH and DVH curves were parameterized using principal component analysis so that noticeable anatomical and dosimetric features were quantified by 1 to 4 principal components with eigenvalue contributions over 97%. For each individual OAR, multivariate regression analysis was carried out to select the variables with statistical significance and thereafter a mathematical model was built using support vector regression (SVR). It was reported that using SVR with a ϵ-insensitive loss function can avoid overfitting and has fewer fitting errors than using multivariable nonlinear regression (9).

As the quality of the plan database may determine the degree of accuracy that a prediction model can offer, a refinement process was performed for the primary model to improve its predictive accuracy (18, 19). This was done by taking the primary model as a self-checking tool and relatively suboptimal database plans were thus identified by comparing the estimated DVHs with the planned DVHs. Unlike previous studies, these suboptimal plans were not excluded from the database, but were rejoined to the training dataset after they were re-optimized by a group of experts under the guidance of the estimated DVHs to further spare the OARs.

The refined model was then used for automatic IMRT planning, by means of which the achievable DVHs were predicted with a 95% confidence interval for each OAR. It is known that the commercial planning system RapidPlan takes the lower bound of the DVH estimate range as the optimization objectives with an attempt to maximize OAR sparing (20). Based on our experience and the previous study (15), we selected the predicted mean value instead of the lower limit of the DVH estimation range as the starting optimization objectives for some adjacent OARs such as the optical chiasm, optical nerve, pituitary, and inner ear in advanced T3-T4 cases to better balance the target dose coverage and normal tissue protection.

### Clinical Evaluation

The clinical test was conducted in 17 new NPC cases of various clinical stages (T1: 2 cases, T2: 1 case, T3: 10 cases, and T4: 4 cases). For each patient, three different IMRT plans were generated: 1) a manual plan (MP): this plan was designed independently by a dosimetrist in the traditional trial-and-error way. 2) A knowledge-based plan (KBP): this plan was automatically generated based on the estimated DVHs by only one click of the ‘optimization’ button with no other human intervention, which is different from the previous study (15). 3) An expert plan (EXP): the MP was adjusted repeatedly by an expert panel with reference to the estimated DVHs until a consensus on the dose distributions was reached. The EXP was regarded as the reference standard in our plan comparison.

In addition, the plan quality variation among planners was investigated by selecting 5 NPC cases of different difficulty (T2: 1 case, T3: 3 cases, and T4: 1 case). For each case, an MP plan was generated independently by three planners with diverse working ages (A: trainee, nearly one-year experience; B: young dosimetrist, three-year experience; and C: senior dosimetrist, more than five-year experience). The resulting plan quality and time consumption were compared.

### Dosimetric Analysis Indices

For a tumor target, a plan comparison was conducted in terms of dose coverage, conformity index (CI), and homogeneity index (HI).

The CI (21) was calculated using the following equation:

$$CI=\left(\frac{V_{Tref}}{V_{T}}\right)*\left(\frac{V_{Tref}}{V_{ref}}\right)$$

where V_Tref_ is the volume of the target covered by the reference isodose, V_T_ is the target volume, and V_ref_ is the volume of the reference isodose.

The HI (22) was defined as:

$$HI=\frac{D_{2\%}-D_{98\%}}{D_{50\%}}$$

where D_x%_ is the absorbed dose received by x% of the target volume.

In this study, 14 kinds of head and neck OARs for NPC treatment were evaluated as shown in **Table 2**. The maximum dose (D_max_ or D_1cc_) and the mean dose (D_mean_) were chosen for the dosimetric evaluation of serial and parallel organs, respectively. The D_1%_ was specially applied for optic organs as their volumes were too small. Other dosimetric indices used are detailed in **Table 2**.

**Table 2:** The quality metric for nasopharyngeal carcinoma cases including the built-in dosimetric indices and the scoring points for tumor targets and organs at risk.

	Parameters	(V:%/D:Gy)	Score
PGTV①	V_98%_	>98/>95/<95	10/6/0
	V_110%_	<10%/<20%/>20%	10/6/0
	CI	>0.8/>0.6/<0.6	10/6/3
	HI	<0.05/<0.10/>0.10	10/6/3
PTV1①	V_98%_	>98/>95/<95	10/6/3
	CI	>0.8/>0.6/<0.6	10/6/3
	HI	<0.05/<0.10/>0.10	10/6/3
PTV2①	V_98%_	>98/>95/<95	10/6/3
	CI	>0.8/>0.6/<0.6	10/6/3
	HI	<0.05/<0.10/>0.10	10/6/3
Brainstem②	D_1cc_	<54/<60/>60	12/6/0
Spinal cord②	D_1cc_	<35/<45/<50/>50	12/6/3/0
Optical chiasm②	D_1%_	<54/<60/>60	12/6/0
Optical nerve②	D_1%_	<54/<60/>60	12/6/0
Temporal lobe②	V_60_	<10/>10	4/2
	D_max_	<60/>60/>65	4/2/0
	D_mean_	<36/>36	4/2
Mandible③	D_max_	<70/<75/>75	5/3/1
TMJ③	D_max_	<70/<75/>75	5/3/1
Parotid③	D_mean_	<26/>26	2/0
	V_50_	<30/>30	3/1
Lens③	D_1%_	<6/<10/>10	5/3/1
Pituitary③	D_1%_	<60/<65/>65	5/3/1
Eye④	D_1%_	<50/>50	1/0
	D_mean_	<35/>35	2/1
Inner-ear④	D_mean_	<50/>50	4/2
Larynx④	D_mean_	<45/>45	4/2
Tongue④	D_mean_	<55/>55	4/2

①tumor targets; ②critical normal organs; ③sub-critical organs; ④other normal organs

To quantify the plan quality, an assessing tool, namely plan quality metric (PQM), was introduced (23). The scoring criteria were established based on our institutional protocols and referenced in the RTOG-0225 and RTOG-0615 guidelines (16, 17) and the work of Ng et al. (24). The total score was 200 points and was divided into 4 levels, i.e., targets (100 points), critical organs (60 points), sub-critical organs (25 points), and other normal organs (15 points). The organ classification and scoring details are listed in **Table 2**.

As for statistical analysis, a Kolmogorov-Smirnov test and homogeneity of variance test were used to affirm the normality and variance homogeneity of the data. For those fulfilling the above two conditions, an F-test was performed or otherwise a Friedman test was applied for a plan comparison. A Bonferroni test was further selected for pair wise comparison in multiple objectives. All statistical analyses were performed using the SPSS software (version 22, SPSS Inc., Chicago, IL).

## Results

### Target Dose Comparison

**Table 3** shows the target dose distribution for three kind of plans. All three groups achieved a dose coverage of V_98%_ higher than 99% for PGTV and PTV1. The hot spot was better controlled in the EXPs (p=0.013), but all three kind of plans had a V_110%_ of lower than 3%. Compared with MPs and EXPs, KBPs acquired increased conformity in PGTV (p<0.001) at the sacrifice of HI in PGTV, PTV1, and PTV2 (p<0.001). It was observed that V_98%_ in PTV2 was significantly lower in KBPs than those in MPs and EXPs (p=0.041).

**Table 3:** Dosimetric and statistical results of tumor targets for the three different plans.

		KBP	MP	EXP	P value	P_1_	P_2_
PGTV	V_98%_ (%)	99.79 ± 0.28	99.70 ± 0.55	99.76 ± 0.32	0.890	–	–
	V_110%_ (%)	2.16 ± 3.12	1.01 ± 2.62	0.51 ± 1.06	0.013	0.030	0.215
	CI	0.53 ± 0.11	0.36 ± 0.15	0.39 ± 0.15	<0.001	<0.001	<0.001
	HI	0.08 ± 0.01	0.07 ± 0.02	0.07 ± 0.02	<0.001	<0.001	0.030
PTV1	V_98%_ (%)	99.05 ± 1.30	99.68 ± 0.56	99.56 ± 0.91	0.003	0.003	0.022
	CI	0.31 ± 0.10	0.31 ± 0.09	0.32 ± 0.10	0.352	–	–
	HI	0.21 ± 0.01	0.19 ± 0.01	0.19 ± 0.01	<0.001	<0.001	<0.001
PTV2	V_98%_ (%)	97.91 ± 2.62	99.28 ± 0.35	98.81 ± 1.39	0.041	0.040	0.283
	CI	0.83 ± 0.02	0.81 ± 0.03	0.82 ± 0.03	0.051	–	–
	HI	0.34 ± 0.02	0.31 ± 0.02	0.32 ± 0.03	0.002	0.002	0.048

P_1_ represents KBP vs MP, P_2_ represents KBP vs EXP.

### OAR Dose Analysis

While the radiation doses to OARs were all managed within the tolerance limits in the three kinds of plans, the KBPs had a slight advantage in OAR sparing than MPs and even EXPs (**Table 4**). Significant dose reduction was achieved in KBPs for critical organs such as the optic chiasm (p<0.001), optic nerve (p=0.021), and temporal lobe (p<0.001), but the KBPs failed to spare the spinal cord compared with MPs and EXPs (p<0.001). As for sub-critical and other normal organs, the KBPs also provided comparable or better protection except for the pituitary (p=0.002) compared with MPs and EXPs.

**Table 4:** Dosimetric and statistical results of organs at risk for the three different plans.

		KBP	MP	EXP	P value	P_1_	P_2_
Brainstem	D_1cc_	50.45 ± 4.85	51.79 ± 4.57	51.07 ± 5.34	0.137	–	–
Spinal cord	D_1cc_	36.98 ± 0.67	35.38 ± 1.89	35.27 ± 1.21	<0.001	<0.001	<0.001
Optic chiasm	D_1%_	42.78 ± 12.24	47.45 ± 15.54	46.26 ± 14.70	0.001	<0.001	0.018
Optic nerve	D_1%_	36.17 ± 18.84	38.53 ± 20.67	37.44 ± 20.07	0.021	0.018	0.51
Temporal lobe	V_60_	2.00 ± 2.65	3.38 ± 3.98	3.09 ± 3.22	<0.001	<0.001	0.005
	D_max_	70.88 ± 6.49	71.15 ± 5.36	71.04 ± 6.25	<0.001	<0.001	<0.001
	D_mean_	17.34 ± 5.65	18.06 ± 5.41	17.90 ± 5.55	0.002	<0.001	0.019
Mandible	D_max_	65.94 ± 7.27	70.68 ± 3.79	69.80 ± 4.17	<0.001	<0.001	<0.001
TMJ	D_max_	61.61 ± 6.18	63.96 ± 7.06	62.82 ± 7.81	0.051	–	–
Parotid	D_mean_	39.65 ± 2.12	40.26 ± 3.04	39.99 ± 2.38	0.310	–	–
	V_50_	33.92 ± 3.15	35.88 ± 5.83	35.43 ± 4.22	0.483	–	–
Lens	D_1%_	6.52 ± 3.13	6.85 ± 3.16	6.71 ± 3.09	0.005	<0.001	0.144
Eye	D_1%_	22.18 ± 9.99	21.56 ± 9.32	21.96 ± 9.68	0.571	–	–
	D_mean_	7.72 ± 3.95	8.21 ± 3.98	8.21 ± 4.32	0.047	0.049	0.044
Pituitary	D_1%_	61.35 ± 9.30	58.77 ± 8.17	59.10 ± 8.00	0.002	<0.001	0.012
Inner-ear	D_mean_	46.62 ± 6.13	47.05 ± 7.84	46.69 ± 8.51	0.580	–	–
Larynx	D_mean_	46.10 ± 2.61	46.22 ± 2.69	45.11 ± 2.56	0.013	1.000	0.044
Tongue	D_mean_	43.12 ± 3.73	44.43 ± 3.99	43.77 ± 4.22	<0.001	<0.001	0.046

P_1_represents KBP vs MP, P_2_ represents KBP vs EXP.

### Overall Plan Quality Evaluation

The plan quality scores are given in **Table 5**. No statistically significant difference was found among the three groups in terms of tumor target (p=0.458), critical organs (p=0.486), sub-critical organs (p=0.225), and other normal organs (p=0.142). The overall plan quality evaluation gave mean scores of 144.59±11.48, 142.71±15.18, and 144.82±15.17, respectively, for KBPs, MPs, and EXPs (p=0.259). A total of 15 out of 17 KBPs (i.e., 88.24%) were approved by our physician as clinically acceptable. In two failure KBP cases, one T3N2 case had extremely low PTV2 coverage (V_98%_=89.75%), and the other, a T4 case, had a very large primary tumor and exhibited unacceptable hot spot areas.

**Table 5:** Plan quality metric scores of the three different plans for 17 nasopharyngeal carcinoma patients.

	KBP	MP	EXP	P value
Critical organs	47.41 ± 5.78	46.35 ± 10.89	47.29 ± 11.66	0.486
Tumor targets	66.24 ± 4.96	66.59 ± 2.55	66.47 ± 2.67	0.458
Sub-critical organs	17.71 ± 3.39	16.76 ± 3.53	17.47 ± 3.71	0.225
Other normal organs	13.24 ± 0.97	13.00 ± 1.22	13.59 ± 1.18	0.142
Total	144.59 ± 11.48	142.71 ± 15.18	144.82 ± 15.17	0.259

The PQM scores varied with different T stages. For relatively easy plans such as T1 and T2 cases, they achieved average scores of 154.00+0.00, 151.33+3.06, and 154.67+2.31, respectively, for KBPs, MPs, and EXPs, which were all the highest scores among the three groups. As for T3 cases, the average PQM scores were 145.20+11.63, 143.60+15.19, and 146.10+14.56 for KBPs, MPs, and EXPs, respectively. For relatively difficult T4 cases, the KBPs, MPs, and EXPs obtained average scores of 141.00+3.61, 143.33+2.31, and 143.33+3.06, respectively.

### Plan Quality Variation Among Planners

The PQM scores of five tested cases were on average 136.60±18.68, 141.40±18.99, and 143.80±20.35, respectively, for dosimetrist A, B, and C (**Table 6**). It was noticed that the plan quality improved with increased experience.

**Table 6:** Plan quality metric scores for three different dosimetrists of varied skill levels, namely A, B, and C.

	Dosimetrist A	Dosimetrist B	Dosimetrist C
Critical normal organsORorgans	42.40 ± 13.67	43.60 ± 14.31	44.80 ± 14.18
Tumor targets	65.80 ± 4.92	66.20 ± 4.49	67.00 ± 3.00
Sub-critical organs	15.40 ± 4.34	18.60 ± 3.29	18.20 ± 4.38
Other normal organs	13.00 ± 1.41	13.00 ± 0	13.80 ± 1.10
Total	136.60 ± 18.68	141.40 ± 18.99	143.80 ± 20.35

**Figure 1:**
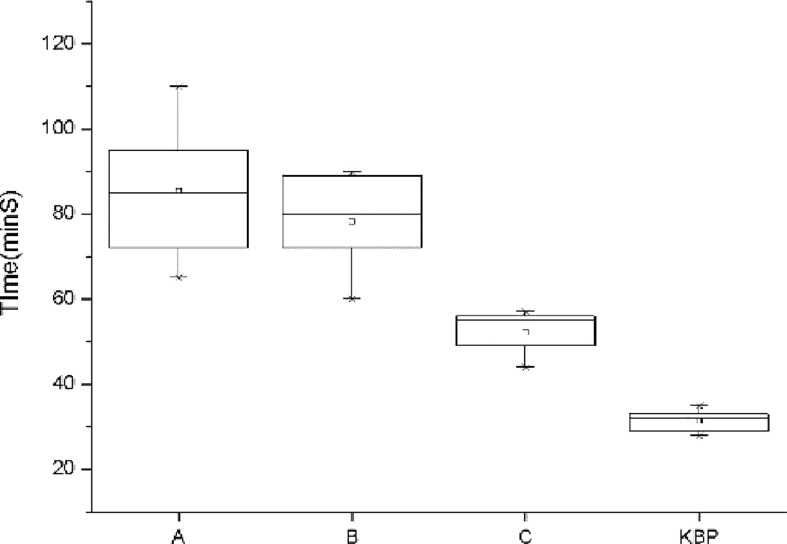
Comparison of the planning time for dosimetrist A, B, C, and auto-planning. The error bar represents 1 standard deviation.

As shown in **Figure 1**, the average time required to achieve clinically acceptable dose distributions decreased with the increase of work experience. However, it was observed that planner C also spent more time than usual in designing the T4 case (55 min). Auto-planning significantly reduced the planning time to within 30 min.

## Discussion

Previously published studies have revealed that quite a few clinical plans may have sub-optimal dose distributions, leading to excessive irradiation to normal tissues (11, 25). KBP methods may provide a possible solution by incorporating prior information into the planning process. In this study, we validated the feasibility and efficiency of a KBP method based on estimated DVHs with special efforts to improve the success rate of auto-planning for NPC treatment. As the database quality might have a direct impact on the prediction results (26), only high quality prior plans with definite curative effects were enrolled. Also, a refinement process was applied here for the primary model to enhance its predictive ability as recommended by several authors (18, 19, 27).

By introducing estimated DVHs, patient-specific optimization objectives rather than general templates were generated for each individual patient in the KBP method, based on the patient anatomy and prior knowledge. This helped offer a clearer directionality for the planner to refine the optimization objectives and achieve a high quality plan, which would be particularly useful for some complicated disease sites such as cancer of the head and neck (28). Our results showed that the EXP method provided the best trade-off between target dose coverage and normal tissue protection, acquiring the highest quality assessment scores among the three kinds of IMRT plans. For T1-T2 and most T3 cases, the KBP method has shown its capability in sparing normal tissues and thus the plan quality score of a fully automatic KBP is better than that of MP, and is close to or reaches the level of EXP. For advanced T4 cases, due to the proximity of neighboring critical structures to the tumor target, some minor improvements in OAR sparing may lead to insufficient target dose coverage, giving the KBP a slightly lower score than MP and EXP. However, no statistically significant difference was found among the three kinds of plans, indicating that the KBP method can produce comparable or even better plans than the traditional manual way. This observation was consistent with previously published studies (15 **,** 28 **,** 29).

It should be noted that we herein applied predicted mean DVH values as the starting optimization objectives for some adjacent OARs such as the optical chiasm, optical nerve, pituitary, and inner ear in advanced T3-T4 cases. This may be the reason why we obtained a higher success rate in auto-planning (about 88%) than the previous study (about 45%) (15). Chang et al. (15) conducted their investigation using a similar estimation module, but took into account the lower bound of the DVH estimate range as the optimization objectives with an attempt to maximize OAR sparing, though the predicted mean usually represents the best estimate from a statistical point of view. For early T1-T2 cases, there is enough distance between tumor targets and the surrounding normal tissues to allow for high dose fall-off, thus relatively “tighter” objectives help achieve better results. However, for advanced T3-T4 cases, applying the lower limit of the estimated DVH as the objective seems too hard to realize for almost all the OARs, especially for the optical chiasm, optical nerve, pituitary, and inner ear which are adjacent to or overlap the target area. These “hard” objectives cause suboptimal trade-off, resulting in insufficient target coverage by the prescribed dose. In fact, even if the predicted mean was selected as the objective, our results demonstrated that the automatic KBP still spared the surrounding critical organs well.

A previously published study applied a scoring system, together with KBP models, to serve as a teaching aid for training IMRT planning skills for lung cancer (30). However, it has been pointed out that this scoring system will always have an ad hoc nature as the preferences of physicians will vary, although the plan scoring system can measure the overall quality of a plan (30). In this study, a similar quality assessment tool was also introduced to quantify the plan quality of NPC cases. The built-in dosimetric indices were referenced in the relevant national and international guidelines, while the scores were given based on our clinical evaluation practice, ensuring that the derived score was in good agreement with the clinical comments. It was shown that for T1 and T2 cases, the high quality plan usually obtained a score of above 150 points, but for T3 and T4 cases, the plan acceptance criteria should be properly reduced to about 140 points. This suggests that if a plan quality score is below these thresholds, for example, if a T2 case obtains an assessing score of less than 150 points, then the planner should be cautious and a systematic quality review would be required to keep the plan standard high. It has been proven to our satisfaction that the quality metric can be calculated within seconds, providing an efficient tool for quick plan quality checks.

However, as shown by us and the previous study (15), the KBP method failed to spare the spinal cord compared with MPs and EXPs. This may be due to the fact that only the primary lesion of the nasopharynx was involved in the DVH prediction model, and the influence of a cervical positive lymph node target was not considered. Recently, Zhang et al. (31) proposed an improved model building method utilizing a so-called generalized distance-to-target histogram to capture the geometric relationships of an OAR with multiple PTVs. This may provide a potential solution for generating a more accurate DVH prediction model for NPC. More research is warranted.

Our results confirmed that traditional manual planning was operator- and experience-dependent. Compared with the junior planner, the experienced dosimetrist was able to produce a high quality plan in a shorter period of time. The KBP method makes full use of prior knowledge, which can generate a plan with quality comparable to that of a senior dosimetrist. However, as commented by Chang et al. (15), the KBP method cannot fully replace the experienced planners, but works more as an aid to guide planners of varied skill levels, especially for the junior planners, to obtain a qualified plan in a more efficient way. By using KBP, the plan quality variation among planners was minimized, thus improving the overall plan quality in a systematic way.

## Conclusions

This study provided evidence that the automatic KBP method can produce clinically acceptable IMRT plans with quality comparable to manual plans for NPC cases. The quality metric helped to quantify the plan quality for a more intuitive evaluation of the planned dose distribution, providing a potential tool for quick plan quality checks.

## Data Availability Statement

The datasets of this research are backed up on the Research Data Deposit (RDD, https://www.researchdata.org.cn, approval number: RDDA2020001752) and are available on reasonable request.

## Ethics statement

Our study was reviewed and approved by the IRB committee of Sun Yat-sen University Cancer Center, with the approval number of B2019-131-01. Written informed consent was obtained from the participants of this study.

## Author Contributions

ZQ designed and supervised the study. ZQ, BL, and JH developed the automatic KBP method. JH, BL, and WX collected and analyzed the data. JZ, XY, HG, MW, and YW provided technical assistance for the study. ZQ, JH, BL and WX wrote the manuscript. All authors contributed to the article and approved the submitted version.

## Funding

This work was supported in part by the National Natural Science Foundation of China, No.81371710; the Science and Technology Program of Guangdong Province, China, No.2013B021800149; and the Science and Technology Program of Guangzhou, China, No.201607010199.

## Conflict of Interest

The authors declare that the research was conducted in the absence of any commercial or financial relationships that could be construed as a potential conflict of interest.
